# Light Rechargeable Lithium-Ion Batteries Using V_2_O_5_ Cathodes

**DOI:** 10.1021/acs.nanolett.1c00298

**Published:** 2021-04-15

**Authors:** Buddha Deka Boruah, Bo Wen, Michael De Volder

**Affiliations:** † Institute for Manufacturing, Department of Engineering, University of Cambridge, Cambridge CB3 0FS, U.K.; ‡ Cambridge Graphene Centre, University of Cambridge, Cambridge CB3 0FA, United Kingdom

**Keywords:** Lithium-ion batteries, V_2_O_5_, photocathodes, photorechargeable batteries

## Abstract

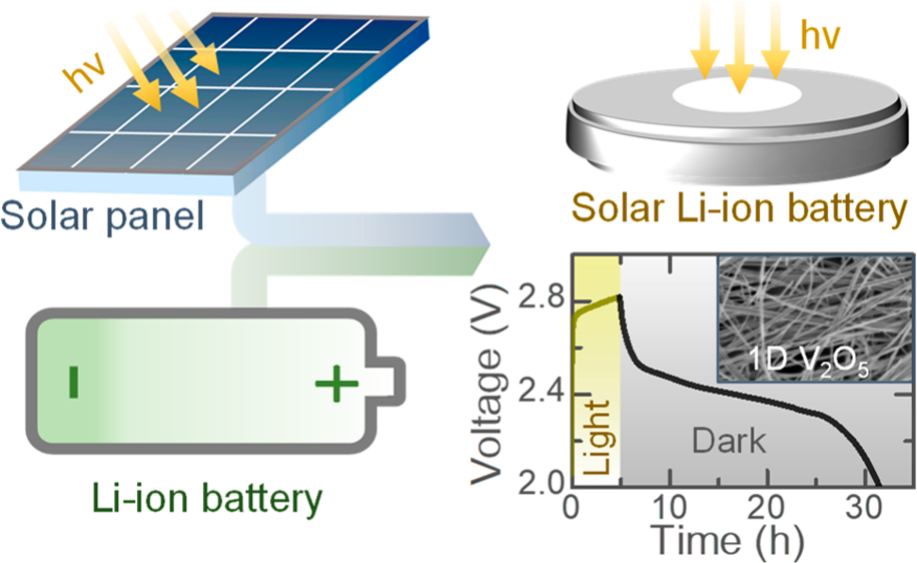

Solar energy is one of the most actively pursued renewable energy sources, but like many other sustainable energy sources, its intermittent character means solar cells have to be connected to an energy storage system to balance production and demand. To improve the efficiency of this energy conversion and storage process, photobatteries have recently been proposed where one of the battery electrodes is made from a photoactive material that can directly be charged by light without using solar cells. Here, we present photorechargeable lithium-ion batteries (Photo-LIBs) using photocathodes based on vanadium pentoxide nanofibers mixed with P3HT and rGO additives. These photocathodes support the photocharge separation and transportation process needed to recharge. The proposed Photo-LIBs show capacity enhancements of more than 57% under illumination and can be charged to ∼2.82 V using light and achieve conversion efficiencies of ∼2.6% for 455 nm illumination and ∼0.22% for 1 sun illumination.

Solar energy has become an important part of our renewable energy production through both large scale solar farms and solar roofs on domestic properties. In addition, energy poverty is a persisting challenge in developing countries, and solar energy is believed to be particularly promising in powering off-grid communities.^1^ However, due to fluctuations in insolation, solar cells, need to be joined up with energy storage devices to balance energy supply and demand.^2,3^ This approach often relies on batteries to store energy, and while a lot of effort has gone in the design of devices that share packaging and current collectors between the solar cells and the batteries to reduce volume, weight, and cost, these solutions often suffer from mismatches between the energy harvesting and storing technology (e.g., operating voltages and manufacturing processes). To address these issues, advanced electrode materials with dual functionalities that can both harvest solar energy similar to a solar cell and at the same time store it similarly to a battery have been proposed. Initially, this work focused on flow batteries,^4^ but recently this expanded to photorechargeable Li-ion batteries (Photo-LIBs) and photocapacitors.^5−8^ Early work on Li-ion batteries (LIBs) suggested a physical mixture of photovoltaic materials and battery materials^6^ and 2D perovskites,^5^ but to date, these systems suffer from limited conversion efficiencies (below 0.1%) or limited life times. More recently, we proposed photorechargeable zinc-ion batteries based on optically and electrochemically active vanadium pentoxide (V_2_O_5_) based photocathodes with improved photoconversion efficiencies of ∼1.2% (455 nm illumination) along with a good cycling stability.^9^ Nevertheless, these batteries are limited in their photocharge output (less than 1 V). In this work, we adapted the above zinc-ion cathodes for Photo-LIBs. We observed conversion efficiencies of ∼2.6% using a 455 nm light source and ∼0.22% for 1 sun illumination, which is an important improvement compared to previous Photo-LIB systems, e.g. 0.034% (420–650 nm illumination) for 2D perovskite LIB^5^ and 0.06% (1 sun illumination) for a LiFePO_4_–Ru dye photocathode-based LIB system.^6^ In addition, photocharging voltages of up to ∼2.82 V within 5 h illumination were achieved along with good cycling stability.

The concept and operation of our Photo-LIBs are depicted in Figure 1a,b. The photocharging capability is obtained by designing photocathodes using one-dimensional (1D) V_2_O_5_ nanofibers mixed with poly(3-hexylthiophene-2,5-diyl) (P3HT) and reduced graphene oxide (rGO), to obtain the required charge separation process (see Figure 1b and discussion further on). The V_2_O_5_ nanofibers used in this work have a typical diameter of 20 to 50 nm (see Figure 1c), and a high-resolution transmission electron microscopy (HRTEM) image of a nanofiber confirms an interplanar spacing of ∼0.34 nm that corresponds to (110) planes (see inset in Figure 1c). 1D V_2_O_5_ nanofibers are used to allow the efficient charge conduction along the nanofiber length.^10^ The nanofibers are synthesized using a process reported previously,^10^ which is summarized in the experimental section (Supporting Information). Figure 1d illustrates energy dispersive X-ray spectroscopy (EDS) mappings of a V_2_O_5_ nanofiber and the representative EDS spectrum shown in Figure S1a. The X-ray powder diffraction (XRD) pattern of V_2_O_5_ nanofibers (Figure 1e) confirmed the orthorhombic V_2_O_5_ crystals structure with a space group of *Pmmn* (59) (JCPDS card no.: 03-065-0131). UV–vis absorption spectrum (Figure 1f) reveals a band edge energy of ∼2.2 eV, and a representative Raman spectrum is provided in the Supporting Information (see Figure S1b).

**Figure 1:**
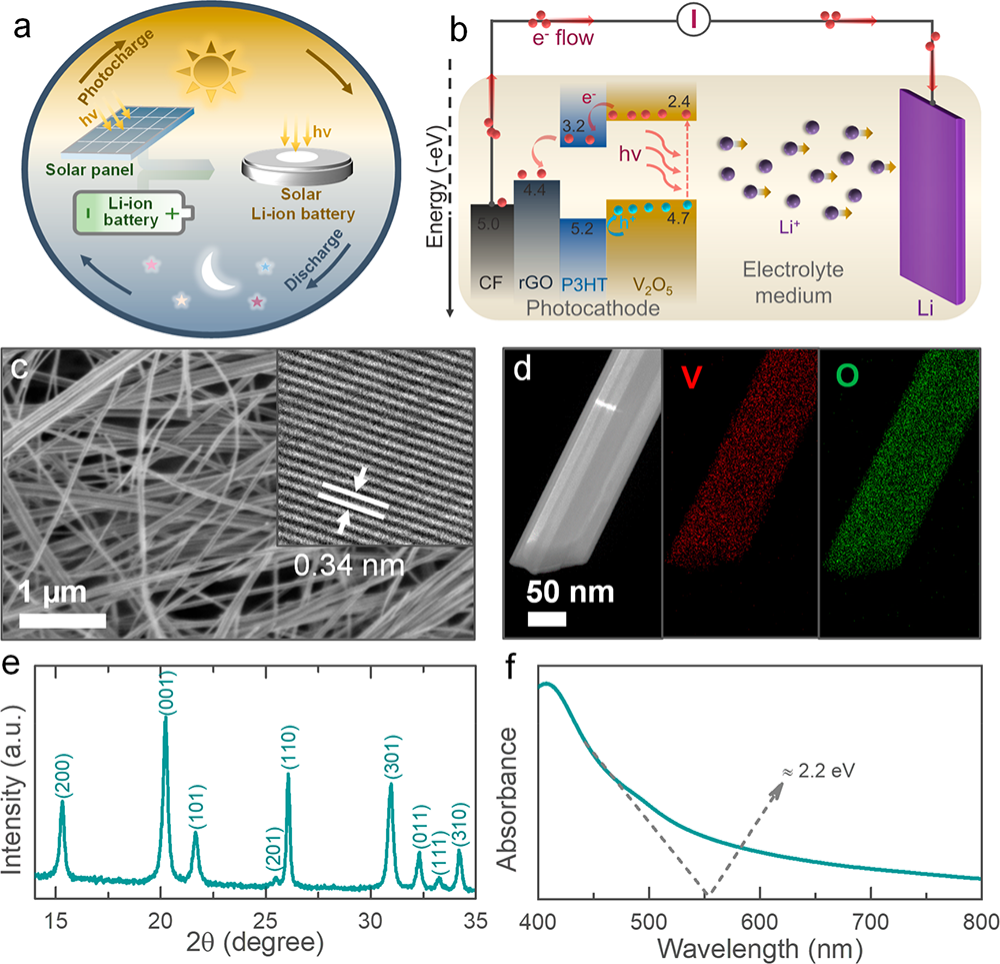
(a) Schematic illustration of our Photo-LIB concept. (b) Schematic representing the photocharging mechanism of Photo-LIB. (c) SEM image of the V_2_O_5_ nanofibers, with the inset showing a HRTEM image. (d) TEM image of a nanofiber and its elemental mappings. (e) XRD pattern of V_2_O_5_ nanofibers that confirms nanofibers have orthorhombic crystal structure with space group of *Pmmn* (59). (f) UV–vis absorption spectrum of the nanofibers with band edge energy of ∼2.2 eV.

Photocathodes are fabricated by mixing 1D V_2_O_5_ nanofibers with P3HT, rGO, and polyvinylidene fluoride (PVDF) in a 93:1:1:5 ratio and drop casted on carbon felt (CF) current collectors (see Experimental Section in the Supporting Information). SEM images of the P3HT, rGO, and photocathode are included in the Supporting Information (see Figure S2). These photocathodes are placed in CR2032 coin cells, in which an ∼7 mm diameter hole is machined and mounted with a transparent glass window to allow for illumination of the photocathodes (see Experimental Section in the Supporting Information). Lithium bis(trifluoromethanesulfonyl)imide (LiTFSI) is used as an electrolyte in this study, using a concentration of 1 or 5 M as discussed further on. All experiments are carried out using a 1 M electrolyte unless specified otherwise.

The energy band diagram of the photocathode configuration is depicted in Figure 1b, where the band energies of P3HT and rGO support the photoexcited electron transportation from V_2_O_5_ nanofibers to the CF current collector. On the other hand, the hole transport from the V_2_O_5_ nanofibers is hindered by the hole blocking properties of P3HT that required effective photocharging of the light rechargeable batteries. We anticipate that the accumulation of electrons on the anode and holes on the cathode drive the Li ion from the photocathode to the anode, thereby effectively recharging the battery. The photosensitivity of V_2_O_5_ and photocharge separation and transportation processes between V_2_O_5_, P3HT and rGO are studied using photodetectors (PDs). Figure 2a shows the current–voltage curves of a planar gold (Au)–V_2_O_5_–Au PD, which demonstrates an increasing current (photocurrent) when illuminated using a light source with energy above the band gap of V_2_O_5_ (Figure S3a, digital image of the PD, Supporting Information). The currents in dark and illuminated states intersected at 0 V, which suggests that an external bias voltage is needed to separate the photogenerated charges from V_2_O_5_. Figure 2b shows the photocurrent in alternating light and dark conditions with 1 V bias voltage to confirm the photocurrent generation under illumination. Next, we fabricated a PD consisting of a fluorine doped tin oxide coated glass (FTO)/rGO/P3HT/V_2_O_5_/Ag stack (see digital image of the PD in Figure S3b). As expected from the energy band diagram in Figure 2d, photocharge separation and transport between V_2_O_5_, P3HT, and rGO result in photocurrent in the absence of external bias voltage, as shown in Figure 2c. Finally, the photocurrent generation without bias voltage, which is important for the photocharging process (see further), is confirmed by current–time measurements under alternating dark and light conditions shown in Figure 2e.

**Figure 2:**
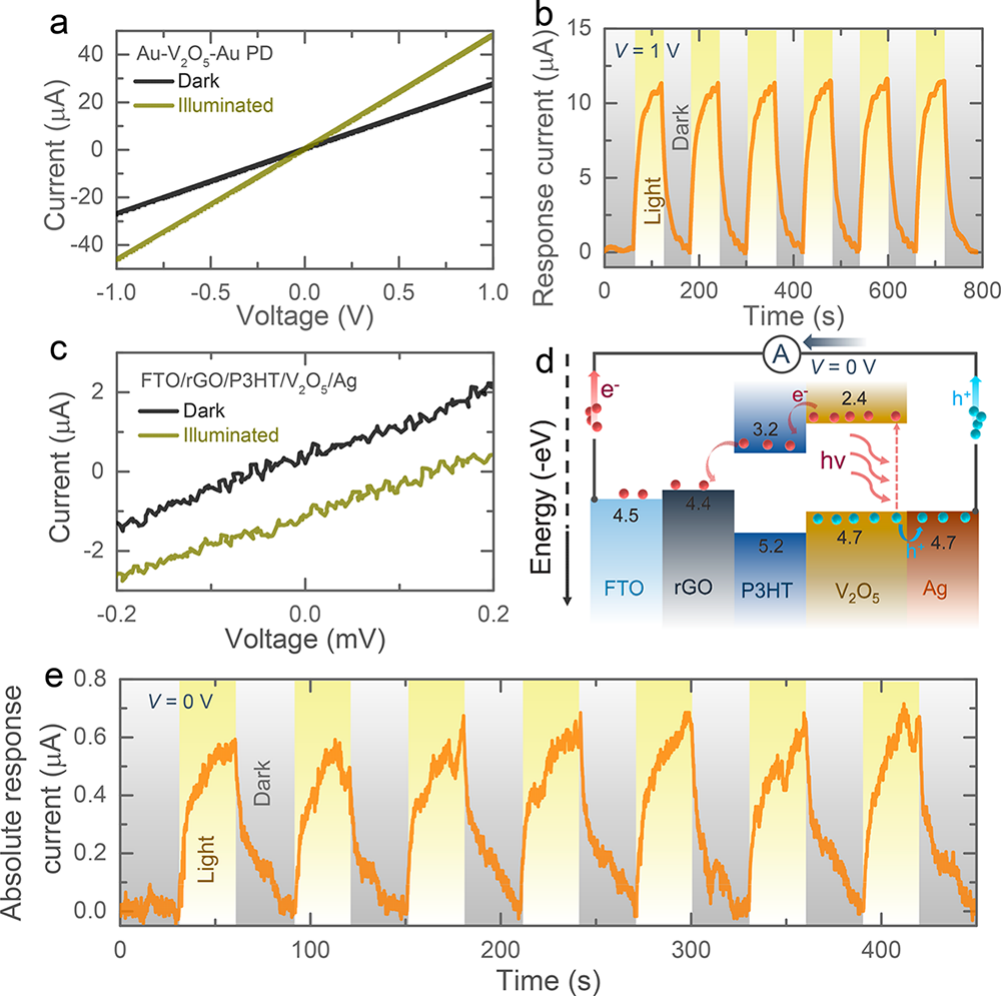
(a) Current–voltage curves of a planar Au–V_2_O_5_–Au PD in dark and illuminated conditions. (b) Response current (= *I*_p_ – *I*_d_, where *I*_d_ and *I*_p_ are the currents in dark and light respectively) of the Au–V_2_O_5_–Au PD under alternating dark and illuminated conditions at 1 V applied bias. (c) Current–voltage curves of a stacked FTO/rGO/P3HT/V_2_O_5_/Ag PD in dark and illuminated conditions. (d) Energy band diagram of the FTO/rGO/P3HT/V_2_O_5_/Ag PD at 0 V. (e) Current–time responses under alternating dark and illuminated states of FTO/rGO/P3HT/V_2_O_5_/Ag PDs at 0 V.

The influence of light on the electrochemical behavior of the proposed Photo-LIBs is first tested using cyclic voltammetry (CV) measurements (scan rates of 0.1–1.0 mV s^–1^, potential window of 2–4 V, light source λ ∼455 nm, intensity ∼12 mW cm^–2^, electrolyte 1 M LiTFSI). The dark CV curves (Figure 3a) at scans of 0.1–1.0 mV s^–1^ show three distinct redox couples with cathodic peaks centered at ∼3.33 V, ∼3.11 V, and ∼2.26 V, corresponding to V_2_O_5_ α-phase transformation to the ε-phase, δ-phase, and γ-phase, respectively along with the respective anodic peaks at ∼2.57 V, ∼3.34 V, and ∼3.53 V.^11^ The reaction mechanisms proposed in literature are^12^
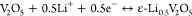

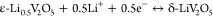

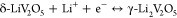


**Figure 3:**
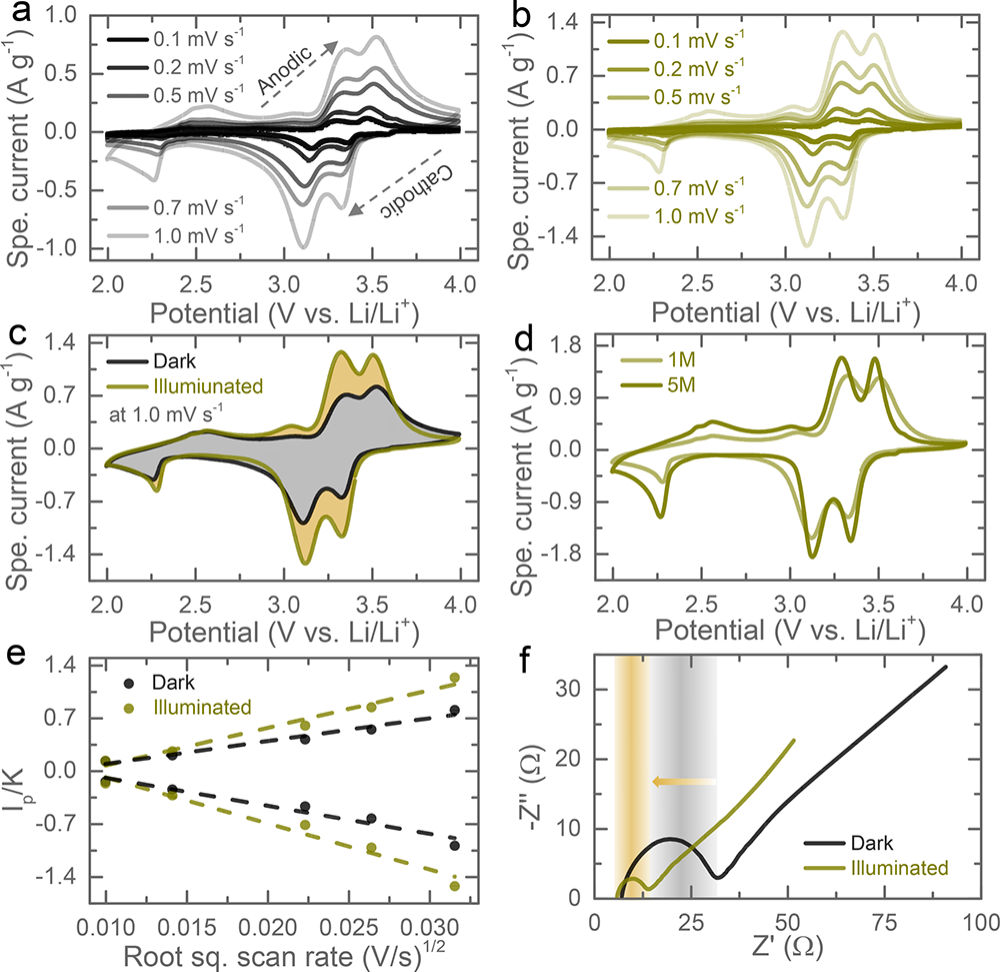
(a,b) CV curves at different scans (0.1 to 1.0 mV s^–1^) between 2 and 4 V in dark and illuminated conditions measured in 1 M LiTFSI. (c) CVs at 1.0 mV s^–1^ in dark and illuminated conditions tested in 1 M LiTFSI. (d) CVs at 1 mV s^–1^ under illumination measured in 1 M LiTFSI and 5 M LiTFSI electrolytes. (e) Diffusion constant analysis in dark and illuminated conditions in 1 M LiTFSI. (f) AC impedance spectra in dark and illuminated conditions (acquired after the 2nd galvanostatic discharge cycle to 3 V with 1 h rest in 1 M LiTFSI electrolyte).

Under illumination, the CV profiles (Figure 3b) roughly retain the same redox peak positions, but the current density increases under illumination. Overall, an ∼30% enhancement in the swept CV area is observed in dark and illuminated conditions as illustrated in Figure 3c (scan rate 1 mV s^–1^). Similar enhancements of ∼30% are observed at scans of 0.5 mV s^–1^ and 0.7 mV s^–1^ in dark and illuminated conditions (see Figure S4). These experiments were repeated with a more concentrated 5 M LiTFSI electrolyte as shown in Figure 3d (CV scan rate of 1 mV s^–1^). These concentrated electrolytes were tested here because they tend to improve the cycling stability as discussed further.^13^ The measurements on concentrated electrolytes in dark conditions are provided in the Supporting Information (see Figure S5).

Next, we study changes in the Li^+^ diffusion constant under light and dark conditions from the peak current densities at cathodic/anodic peaks at ∼3.11 V/∼3.53 V at different CV scan rates. The diffusion constant (*D*) is calculated from^14^
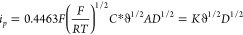
where *i*_*p*_, C*, ϑ, *F*, and *A* represent peak current, initial concentration in mol cm^–3^, scan rate in V s^–1^, Faraday constant, and electrode area in cm^2^ respectively and C*A. The active electrode area is difficult to measure, but if we assume this does not change by exposing the electrode to light, we can calculate the relative increase in diffusion constant, which we found to be ∼65% and ∼64.4% for cathodic and anodic reactions at ∼3.11 V/∼3.53 V based on the slopes of the ϑ^1/2^ vs *i*_*p*_/*K* (see Figure 3e). Electrochemical Impedance Spectroscopy (EIS) measurements carried out between 10 mHz and 100 kHz at a voltage amplitude of 10 mV are shown in Figure 3f. These measurements suggest a small decrease in combined series resistance and charge transfer resistance from ∼32 Ω to 14 Ω under illumination, which is in accordance with previous reports on photobatteries.^7,9^ The decrease in the charge transfer resistance of the photocathode under illumination could be due to the increase in charge carrier density resulting in better electrical conductivity. The equivalent circuit used to analyze the EIS data is provided in Figure S6.

Figure 4a,b show galvanostatic discharge–charge measurements at specific currents of 200 mA g^–1^ and 500 mA g^–1^ in light and dark conditions. These measurements show increases in capacity from ∼118 mAh g^–1^ (∼0.0944 mAh cm^–2^) to ∼161 mAh g^–1^ (∼0.1288 mAh cm^–2^) and ∼81 mAh g^–1^ to ∼127 mAh g^–1^ and nominal voltages from 2.88 to 2.92 V and 2.78 to 2.86 V respectively under illumination. Note that, under illumination, the photocurrent generated in the electrode will accrue a certain amount of mAh over time, which shows up as an additional capacity in our galvanostatic measurements.^15^ For completeness, Figure S7 illustrates comparative discharge–charge curves at specific currents of 100 mA g^–1^, 1000 mA g^–1^, and 2000 mA g^–1^. d*Q*/d*V* plots (Figure 4c) show a similar increase in current density of redox peaks under illuminated conditions as the CV measurements in Figure 3c. The rate tests in light and dark conditions of the Photo-LIBs confirmed that, even at high specific current densities of 2000 mA g^–1^, Photo-LIBs still retain an increase in capacity under illumination. Furthermore, discharge–charge curves of the Photo-LIBs tested in 5 M LiTFSI electrolyte are reported in Figure S8. Prolonged cycling tests of the Photo-LIBs in 1 and 5 M LiTFSI electrolytes at a specific current of 300 mA g^–1^ show a rapid capacity decay at low concentrated (1 M) electrolyte in the first 25 cycles after which the capacity fade stabilizes for the following 175 discharge–charge cycles (Figure 4e). Further, *ex situ* measurements show that the Raman spectra of the photocathodes are reversible (see Figure S9). Using the concentrated 5 M electrolyte slows the capacity fade initially, but after 75 cycles, the capacity of the 1 and 5 M batteries is similar. However, the photocharge voltage response is better when using a 1 M electrolyte (see further).

**Figure 4:**
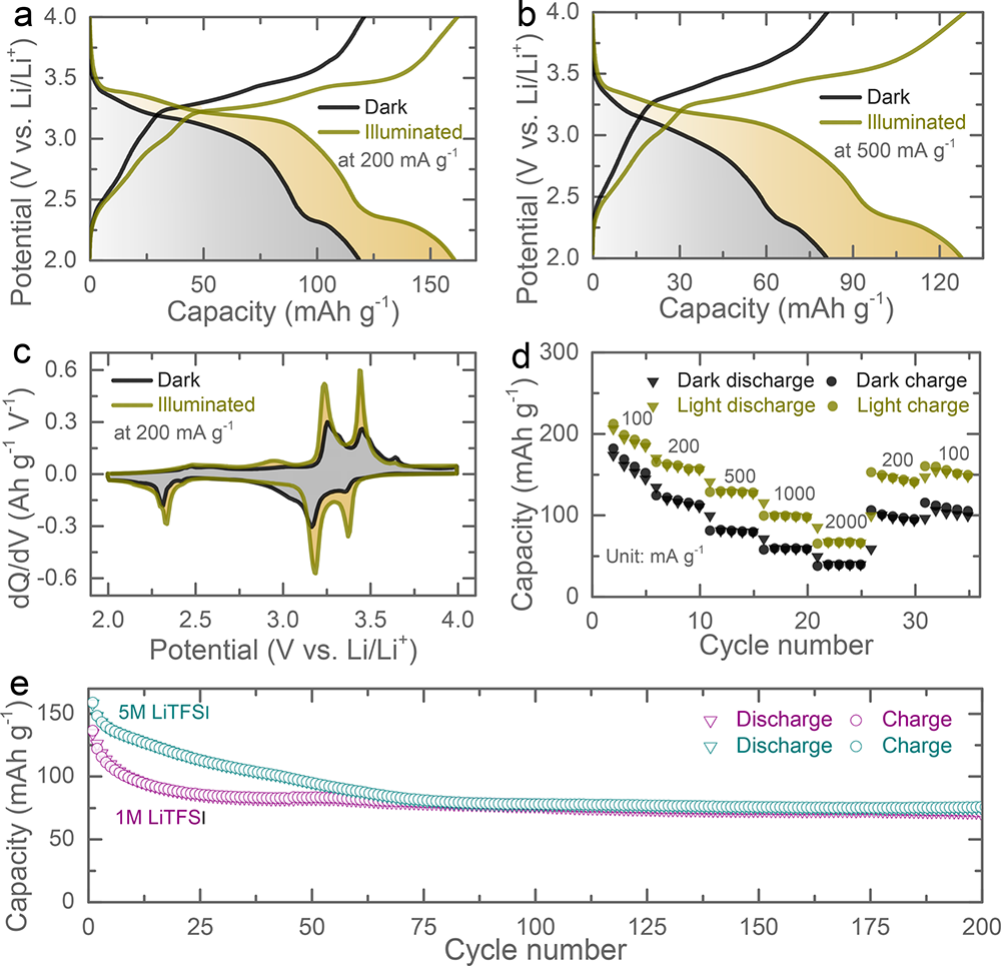
(a,b) Galvanostatic discharge–charge curves at 200 mA g^–1^ (7th cycle) and 500 mA g^–1^ (12th cycle) in dark and illuminated conditions. (c) d*Q*/d*V* curves of the respective discharge–charge curves at 200 mA g^–1^. (d) Rate performance tests of the Photo-LIBs in dark and illuminated conditions. (e) Cycling stability (at 300 mA g^–1^) of the Photo-LIBs tested in 1 and 5 M LiTFSI electrolytes in dark conditions.

Both the CV (Figure 3) and galvanostatic (Figure 4) experiments discussed above indicate a difference in the light-electrode interaction as a function of the state of charge (e.g., the current enhancement changes with the state of charge), which might for instance result from changes in the band gap of the cathode material as it is lithiated. Therefore, *in situ* and *ex situ* UV–vis measurements of the photocathodes are conducted to understand how the optical properties of the cathodes change as a function of the state of charge. For these tests, we drop casted photocathodes on transparent FTO coated glass substrates as FTO does not undergo lithiation until well below 1.5 V vs Li/Li^+^ and mount these in optical cells.^5,16,17^ As shown in Figure 5a, the color of the photocathode changes in discharge and charge states—the orangelike photocathode color changes to light gray while discharged to 2 V and returns to its original color when charged to 4 V (see images in Figure 5b). This color change is quantified by mounting an optical battery cell in a UV–vis integrating sphere (see Experimental Section in the Supporting Information) and measuring reflectance spectra of the photocathode *in situ*. Figure S10 shows the reflectance spectrum of the photocathode changing as a function of the state of charge, which indicates changes in the band gap^18^ and therefore also in the light-charging properties. To measure the optical band gap energy of the V_2_O_5_ photocathode more accurately, we measure the UV–vis absorption of the photocathodes *ex situ* (see Figure 5c). This measurement shows that insertion of Li^+^ into V_2_O_5_ increases the band gap (shifts absorption peak toward lower wavelengths), and this process is reversible when extracting Li^+^ from V_2_O_5_. This band gap shift was reported previously for the α-phase transforming in the ε-phase.^19^ It is noted that insertion of Li^+^ into V_2_O_5_ moves up the Fermi level near the split-off band and widens the optical band gap (*E*_*g*_) followed by the relation of *E*_*g*_ = *E*_*g*0_ + *E*_*g*1_, where *E*_*g*0_ represent the optical band gap of the V_2_O_5_ before Li^+^ insertion (pristine V_2_O_5_) and *E*_*g*1_ represents the Burstein–Moss (BM) shift.^18,20^

**Figure 5:**
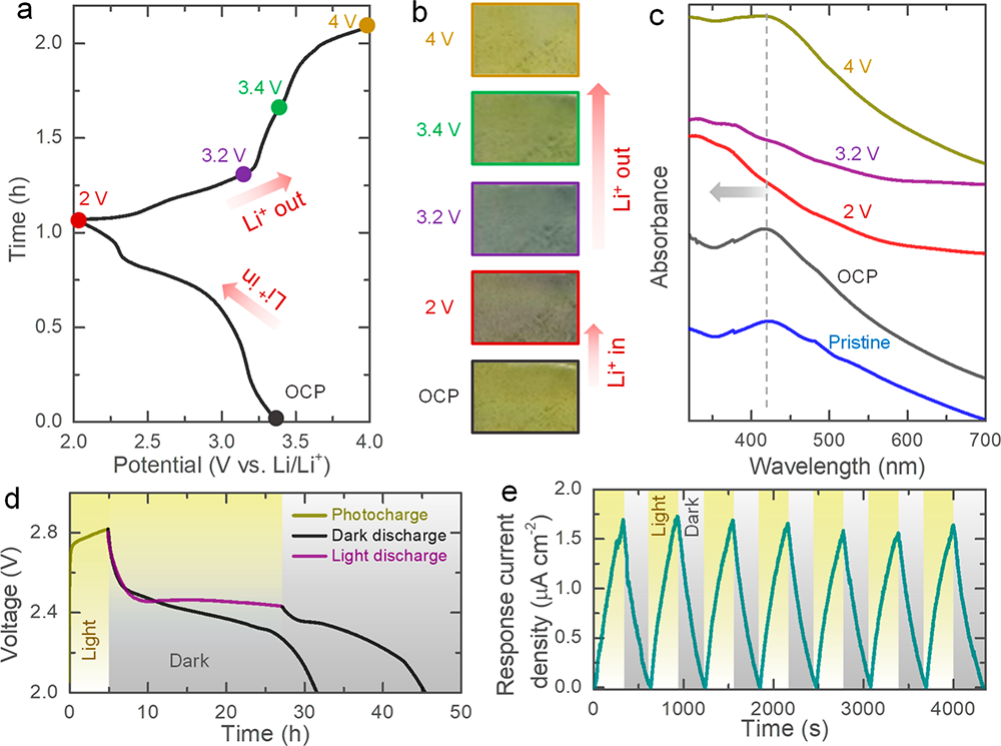
(a, b) Initial discharge–charge curve using an optical-cell along with the images showing the change in color as a function of the state of charge. (c) Ex situ absorbance spectra of the photocathodes at different states of charge. (d) Photocharge for 5 h and galvanostatic discharge at 200 mA m^–2^ in dark and illuminated conditions. (e) Variation of absolute response current density of the Photo-LIB under periodic dark and light illuminated states at 0 V applied voltage.

In what follows, the light charging capabilities of the Photo-LIBs are measured without applying any external current. After light charging, the battery is discharged galvanostatically in either light or dark. As shown in Figure 5d, the voltage increases to ∼2.82 V when illuminated for 5 h (λ ∼455 nm, intensity ∼12 mW cm^–2^), and this increases to ∼3.0 V after prolonged illumination (see Figure S11a). Under illumination, the photogenerated electrons are transported from the photocathode to the Li counter electrode through the external circuit. We assume that the photogenerated holes (*h*^+^) help drive the charging process by changing the oxidation state of vanadium and release the Li^+^ (Li_*x*_V_2_O_5_ + *yh*^+^ → *y*^Li+^ + Li_*x*–*y*_V_2_O_5_). At the same time the photogenerated electrons transported to the Li counter electrode will reduce the Li^+^ ion (Li^+^ + *e*^–^ → Li). Figure 5d also shows the discharge profiles under light and dark conditions. In the specific conditions of this experiment, the balance between charge and discharge results in a nearly constant ∼2.45 V voltage response. Once the light is turned off, the Photo-LIBs discharge to the cutoff voltage of 2 V as expected from the dark discharge response. The discharge responses at different specific currents of the photocharged Photo-LIBs in dark are provided in the Supporting Information (see Figure S11b). In addition, Photo-LIBs charged only by light can power commercial sensors (1.5 V Digital Thermo-Hygrometer TFA, MPN: 30.5005) and green LEDs (2 V LED) as shown in Figure S12a,b.

Chronoamperometry measurements (Figure 5e) of our Photo-LIBs in dark and light conditions show consistent increase in the absolute response current of 0 to ∼1.7 μA cm^–2^ in dark and light, respectively. Further, it is observed that with increasing electrolyte concentration the photocharging voltage decreases (Figure S13a), which may in part be due to the higher light absorption of the 5 M electrolytes shown in the UV–vis spectra in Figure S13b. Our Photo-LIBs can also be charged by sunlight (400–1100 nm, LED Solar Simulator LSH-7320), however, since some part of the solar spectrum has a photon energy lower than the required band gap energy for photocharging and hence the charging times are longer than for the above 455 nm light sources (see Figure S13c). Further, electrodes without P3HT have a lower voltage response as demonstrated in Figure S14. This is probably due to a combination of the hole blocking properties of P3HT as well as its ability to generate additional electron hole pairs. While the combination of V_2_O_5_ and P3HT gave promising results in our experiments, we anticipate that other combinations of cathode materials with a band gap that aligns with the solar spectrum in conjunction with hole blocking layers should be able to create light charging batteries. Finally, the photoconversion efficiency of the presented Photo-LIBs is ∼2.6% for 455 nm illumination and ∼0.22% for 1 sun illumination (see calculation in the Supporting Information), which is higher than the previously reported photoconversion efficiencies of 0.03 to 0.06% for Photo-LIBs^5,6^ as well as photorechargeable zinc-ion batteries with efficiencies ranging from 0.18% to 1.2% for 455 nm illumination.^9,21^ Compared to the latter, the current LIB system also achieves a substantial improvement in the average output voltage, from 0.77 V for ZIBs to 2.88 V for the LIBs proposed here, which is an important step forward for photobattery applications.

This work presents a new LIB that can be charged directly by light without the need for external power sources or solar cells. This is achieved by designing a photocathode that can store Li ions as well as create and separate photocharge carriers. These Photo-LIBs show capacity enhancements of more than 57% under illumination and reach up to ∼2.82 V under light illumination for 5 h (λ ∼455 nm, intensity ∼12 mW cm^–2^). Light conversion efficiencies of up to ∼2.6% for 455 nm illumination and ∼0.22% for 1 sun illumination have been achieved, which is higher than other systems reported so far.

## Supporting Information Available

Preparation of photocathodes; Material characterization; designing of Photo-LIBs; Electrochemical characterization of Photo-LIBs; In situ optical characterizations; Ex situ UV–vis and Raman characterizations; Fabrication of PDs and Electrical Measurements; EDS and Raman spectrum of V_2_O_5_ nanofibers (Figure S1); SEM images of P3HT, rGO and photocathode (Figure S2); digital images of the PDs (Figure S3); comparative CV curves in 1 M LiTFSI electrolyte in dark and light (Figure S4); comparative CVs of Photo-LIBs in 5 M LiTFSI in dark and illuminated (Figure S5); equivalent circuit for the Nyquist plots (Figure S6); comparative discharge–charge curves in dark and light tested in 1 M LiTFSI (Figure S7) and 5 M LiTFSI (Figure S8); *ex situ* Raman spectra of the photocathodes (Figure S9); *in situ* optical reflectance spectra (Figure S10); long time photocharging response (Figure S11a); photocharge and discharges at different specific currents tested in 1 M LiTFSI (Figure S11b); digital photographs of powering a Thermo-hygrometer and LED (Figure S12); Photocharges and discharges with different LiTFSI electrolyte concentrations (Figure S13a); absorption spectra of the 1 and 5 M LiTFSI electrolytes (Figure S13b); photocharges under 1 Sun and 455 nm illuminations (Figure S13c); CVs of P3HT free V_2_O_5_-rGO photocathodes based Photo-LIBs in 5 M LiTFSI electrolyte (Figure S14); photocharges of the Photo-LIBs based on P3HT free V_2_O_5_-rGO and V_2_O_5_ P3HT-rGO photocathodes in 5 M LiTFSI electrolyte (Figure S14d); SEM, XRD, and Raman of commercial V_2_O_5_ powder (Figure S15) (PDF)

## References

[ref1] The World Bank, Report PAD2635, 2019.

[ref2] Zeng Q.; Lai Y.; Jiang L.; Liu F.; Hao X.; Wang L.; Green M. A. Integrated Photorechargeable Energy Storage System: Next-Generation Power Source Driving the Future. Adv. Energy Mater. 2020, 10, 1903930 10.1002/aenm.201903930.

[ref3] Gurung A.; Qiao Q. Solar Charging Batteries: Advances, Challenges, and Opportunities. Joule 2018, 2, 1217 10.1016/j.joule.2018.04.006.

[ref4] Paolella A.; Vijh A.; Guerfi A.; Zaghib K.; Faure C. Review-Li-Ion Photo-Batteries: Challenges and Opportunities. J. Electrochem. Soc. 2020, 167, 120545 10.1149/1945-7111/abb178.

[ref5] Ahmad S.; George C.; Beesley D. J.; Baumberg J. J.; De Volder M. Photo-Rechargeable Organo-Halide Perovskite Batteries. Nano Lett. 2018, 18, 1856 10.1021/acs.nanolett.7b05153. 29425044

[ref6] Paolella A.; Faure C.; Bertoni G.; Marras S.; Guerfi A.; Darwiche A.; Hovington P.; et al. Light-assisted delithiation of lithium iron phosphate nanocrystals towards photo-rechargeable lithium ion batteries. Nat. Commun. 2017, 8, 14643 10.1038/ncomms14643. 28393912 PMC5394232

[ref7] Boruah B. D.; Wen B.; Nagane S.; Zhang X.; Stranks S. D.; Boies A.; De Volder M. Photo-rechargeable Zinc-Ion Capacitors using V2O5-Activated Carbon Electrodes. ACS Energy Lett. 2020, 5, 3132 10.1021/acsenergylett.0c01528.

[ref8] Boruah B. D.; Mathieson A.; Wen B.; Jo C.; Deschler F.; De Volder M. Photo-Rechargeable Zinc-Ion Capacitor Using 2D Graphitic Carbon Nitride. Nano Lett. 2020, 20, 5967 10.1021/acs.nanolett.0c01958. 32589038

[ref9] Boruah B. D.; Mathieson A.; Wen B.; Feldmann S.; Dose W. M.; De Volder M. Photo-rechargeable zinc-ion batteries. Energy Environ. Sci. 2020, 13, 2414 10.1039/D0EE01392G.

[ref10] Zhai T.; Liu H.; Li H.; Fang X.; Liao M.; Li L.; Zhou H.; Koide Y.; Bando Y.; Golberg D. Centimeter-long V2O5 nanowires: from synthesis to field-emission, electrochemical, electrical transport, and photoconductive properties. Adv. Mater. 2010, 22, 2547 10.1002/adma.200903586. 20449845

[ref11] Palanisamy K.; Um J. H.; Jeong M.; Yoon W.-S. Porous V2O5/RGO/CNT hierarchical architecture as a cathode material: Emphasis on the contribution of surface lithium storage. Sci. Rep. 2016, 6, 31275 10.1038/srep31275. 27511434 PMC4980635

[ref12] Chao D.; Xia X.; Liu J.; Fan Z.; Ng C. F.; Lin J.; Zhang H.; Shen Z. X.; Fan H. J. A V2O5/conductive-polymer core/shell nanobelt array on three-dimensional graphite foam: a high-rate, ultrastable, and freestanding cathode for lithium-ion batteries. Adv. Mater. 2014, 26, 5794 10.1002/adma.201400719. 24888872

[ref13] Wang J.; Yamada Y.; Sodeyama K.; Watanabe E.; Takada K.; Tateyama Y.; Yamada A. Fire-extinguishing organic electrolytes for safe batteries. Nat. Energy 2018, 3, 22 10.1038/s41560-017-0033-8.

[ref14] Yu D. Y. W.; Fietzek C.; Weydanz W.; Donoue K.; Inoue T.; Kurokawa H.; Fujitani S. Study of LiFePO4 by Cyclic Voltammetry. J. Electrochem. Soc. 2007, 154, A253 10.1149/1.2434687.

[ref15] Kato K.; Puthirath A. B.; Mojibpour A.; Miroshnikov M.; Satapathy S.; Thangavel N. K.; Mahankali K.; et al. Light-Assisted Rechargeable Lithium Batteries: Organic Molecules for Simultaneous Energy Harvesting and Storage. Nano Lett. 2021, 21, 907 10.1021/acs.nanolett.0c03311. 33416335

[ref16] Xu H.; Shi L.; Wang Z.; Liu J.; Zhu J.; Zhao Y.; Zhang M.; Yuan S. Fluorine-Doped Tin Oxide Nanocrystal/Reduced Graphene Oxide Composites as Lithium Ion Battery Anode Material with High Capacity and Cycling Stability. ACS Appl. Mater. Interfaces 2015, 7, 27486 10.1021/acsami.5b09538. 26606370

[ref17] Nguyen O.; Courtin E.; Sauvage F.; Krins N.; Sanchez C.; Laberty-Robert C. Shedding light on the light-driven lithium ion deinsertion reaction: towards the design of a photorechargeable battery. J. Mater. Chem. A 2017, 5, 5927 10.1039/C7TA00493A.

[ref18] Wang Q.; Brier M.; Joshi S.; Puntambekar A.; Chakrapani V. Defect-induced Burstein-Moss shift in reduced V2O5 nanostructures. Phys. Rev. B: Condens. Matter Mater. Phys. 2016, 94, 245305 10.1103/PhysRevB.94.245305.

[ref19] Tolhurst T. M.; Leedahl B.; Andrews J. L.; Banerjee S.; Moewes A. The electronic structure of ε′-V2O5: an expanded band gap in a double-layered polymorph with increased interlayer separation. J. Mater. Chem. A 2017, 5, 23694 10.1039/C7TA05066F.

[ref20] Wu G.; Du K.; Xia C.; Kun X.; Shen J.; Zhou B.; Wang J. Optical absorption edge evolution of vanadium pentoxide films during lithium intercalation. Thin Solid Films 2005, 485, 284 10.1016/j.tsf.2005.03.039.

[ref21] Boruah B. D.; Mathieson A.; Park S. K.; Zhang X.; Wen B.; Tan L.; Boies A.; Volder M. D. Vanadium Dioxide Cathodes for High-Rate Photo-Rechargeable Zinc-Ion Batteries. Adv. Energy Mater. 2021, 11, 2100115 10.1002/aenm.202100115.

