# Supplementary material for: Light Rechargeable Lithium-Ion Batteries Using V2O5 Cathodes
Source: Nano Lett. 2021 Apr 15;21(8):3527–32. doi: 10.1021/acs.nanolett.1c00298 (PMC8155332; doi:10.1021/acs.nanolett.1c00298)
Supplement: Supplementary file 1 — nl1c00298_si_001.pdf [file nl1c00298_si_001.pdf]

Supporting Information

for

Light Rechargeable Lithium-ion Batteries Using V_2O_5 Cathodes

Buddha Deka Boruah[1], Bo Wen[1,2], Michael De Volder[1,*]

[1]Institute for Manufacturing, Department of Engineering, University of Cambridge, Cambridge CB3 0FS, UK

[2]Cambridge Graphene Centre, University of Cambridge, Cambridge CB3 0FA, United Kingdom

*Corresponding author. E-mail: mfld2@cam.ac.uk

Experimental Section

Preparation of Photocathodes: The V_2O_5 nanofibers were synthesized followed by a hydrothermal process, where 0.728 g commercial V_2O_5 powder (received from Sigma-Aldrich) added in 60 mL de-ionized (DI) water mixed followed by stirring. Thereafter, 10 mL of 30% H_2O_2 (Sigma-Aldrich) added and stirred until obtaining a transparent orange solution. Finally, the solution was transferred into an autoclave and maintained at 205 ^{o}C for two days. As obtained product was centrifuged using DI water and ethanol. Finally, the V_2O_5 nanofibers obtained by annealing at 400 ^{o}C in air. The SEM, XRD, Raman of the used commercial V_2O_5 powder provided in the Supporting Information (see **Figure S15**).

Then, 91 mg V_2O_5 nanofibers, 2 mg P3HT and 2 mg rGO dispersed in 4 mL N-Methyl-2-pyrrolidone (NMP, Sigma-Aldrich) using sonication process. Then, the electrode solution obtained by adding 5 mg polyvinylidene fluoride (PVDF, Solef 6020) binder. Finally, the photocathodes are obtained by direct drop casting the electrode solution on CF (Sigracet GDL 39 AA carbon graphite paper, SGL Carbon). P3HT free photocathodes are prepared by mixing 91 mg V2O5 nanofibers, 4 mg rGO solution and 5 mg PVDF binder.

Material Characterization: The morphologies and elemental analysis of the samples are characterized by SEM (FEI Magellan 400L) and TEM (Talos F200X G2). Crystal structures are analysed by XRD (Bruker D8 Advance, Cu Kα radiation). Further, Raman spectroscopy (Renishaw InVia) and UV/VIS/NIR Spectrometer (Lamda 750) are used for characterising for optical properties.

Designing of Photo-LIBs: Coin cell (CR2032) type Photo-LIB designed by making a ~ 7 mm hole (diameter) on coin cell case sealed with a transparent glass window using EPOXY (EVO-STIK) for light illumination. Then photocathode is placed and connected using aluminum strip between electrode and coin cell for electrical connectivity. Thereafter, Whatman glass

microfiber filters paper separators are placed on the photocathode by adding ~ 60 μL LiTFSI in EC/PC (1:1) electrolyte. Finally, Li counter electrodes are placed and assembled in Photo-LIBs following standard procedures.

Electrochemical Characterization of Photo-LIBs: First, CV curves of the Photo-LIBs at different scan rates (0.1 to 1.0 mV s^{-1}) over the potential window of 2 V – 4 V in dark and illuminated (light source λ ~ 455 nm, intensity ~ 12 mW cm^{-2}) conditions were measured by using a Biologic VMP-3 galvanostat. Galvanostatic discharge-charge tests of the Photo-LIBs are recorded at different specific currents (100 to 2000 mA g^{-1}) in dark and illuminated conditions. AC impedance (EIS) tests recorded in the frequency range from 10 mHz to 100 kHz at voltage amplitude of 10 mV in dark and illuminated conditions. Further, photocharge responses of the Photo-LIBs are tested by recording open circuit voltage in absence of external current under illumination and discharged by applying specific currents.

In-situ Optical Characterizations: To understand the optical properties of the photocathodes, *in-situ* reflectance spectra were measured at different stages of discharge and charge at a specific current using PerkinElmer UV/Vis/NIR Spectrometer (Lamda 750) in reflection mode.

Ex-situ UV-Vis and Raman Characterizations: To measure the *ex-situ* UV-Vis absorption spectra of the photocathodes at different discharge and charge states, the photocathodes are prepared by drop casting on the FTO coated glass substrates (surface resistivity ~7 Ωsq^{-1}, Sigma-Aldrich) and assembled optical-cells followed by the procedure reported in our previous work.[1] Theses optical-cells are then cycled to different states of discharge and charge under constant current mode. As cycled optical-cells were disassembled and washed with PC. The optical absorption spectra of these photocathodes were measured using a PerkinElmer UV/Vis/NIR Spectrometer (Lamda 750). Likewise, Raman spectra of these photocathodes characterized by using a Renishaw InVia to understand the charge storage reversibility.

Fabrication of PDs and Electrical Measurements: The electrical photoresponse of V_2O_5 was measured by patterning Au/chromium (Cr) (40/10 nm) IDEs on a Si_3N_4/Si wafer using UV lithography, then V_2O_5 cast on the IDEs followed by drying at 120 ºC in vacuum oven. Then, current – voltage measurements are recorded by sweeping the voltage from -1 V to +1 V in dark and illuminated conditions. Moreover, current – time tests under alternative dark and illuminated conditions were recorded in presence (V = 1 V) of external bias voltage. Likewise, the stacked FTO/rGO/P3HT/V_2O_5/Ag PD was fabricated followed by layer-by-layer coating of materials on the FTO coated glass substrate. Finally, Ag paste contact was used to measure current – voltage in dark and illuminated conditions as well as current – time tests under alternative dark and illuminated conditions in absence of external bias voltage (V = 0 V).

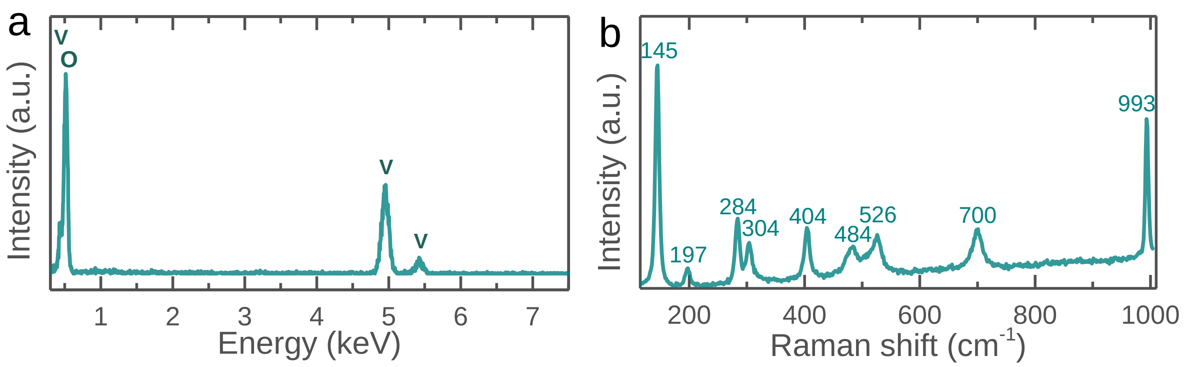

Figure S1. *(a) EDS spectrum of a V_2O_5 nanofiber (**Figure 1d**). (b) Raman spectrum of as synthesized V_2O_5 nanofibers.*

Figure S1a shows the EDS spectrum of a single V_2O_5 nanofiber (**Figure 1d**), where the Raman spectrum of the V_2O_5 nanofibers shown in **Figure S1b**. The characteristic Raman shifts centered at around ~ 993 cm^{-1} belongs to stretching vibration of vanadyl bond (V = O) bond, ~ 700 cm^{-1} belongs to stretching of V – O – V bonds, ~ 526 cm^{-1} corresponds to V_3O phonon band, 484 cm^{-1} assigns to V – $O_{(3)}$ – V symmetric stretching, ~ 404 cm^{-1} represents to angle-bending of V – $O_{(3)}$ – V, ~ 304 cm^{-1} belongs to bending vibration of V – O_C, ~ 284 cm^{-1} belongs to bond bending vibration of O_C – V – O_B, ~ 197 cm^{-1} belongs to bending vibration of O_C – V – O_B (A_g mode) bond and ~ 145 cm^{-1} corresponds to vibration mode of V – O – V chains.[2-4]

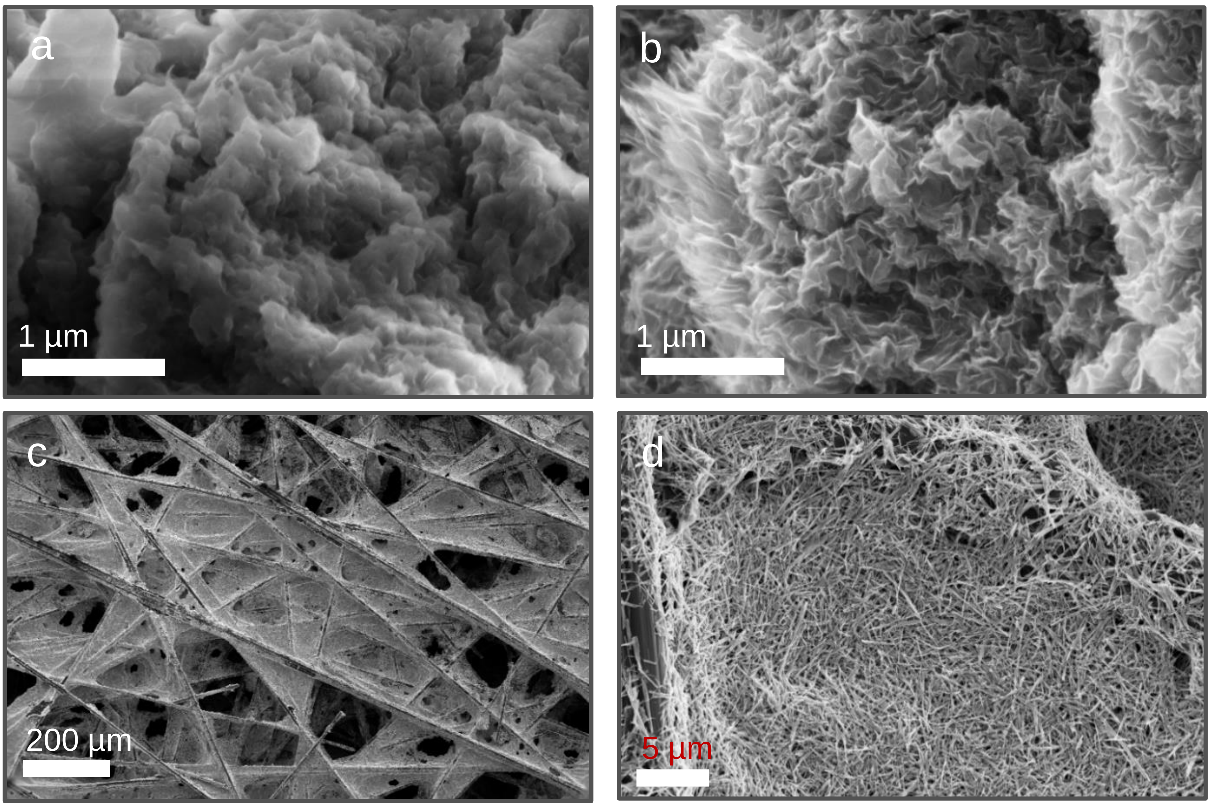

Figure S2. SEM images of (a) P3HT, (b) rGO and (c,d) photocathode at low and high magnifications.

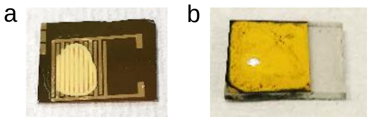

Figure S3. *Digital images of (a) Au-V_2O_5-Au and (b) FTO/rGO/P3HT/V_2O_5/Ag PDs.*

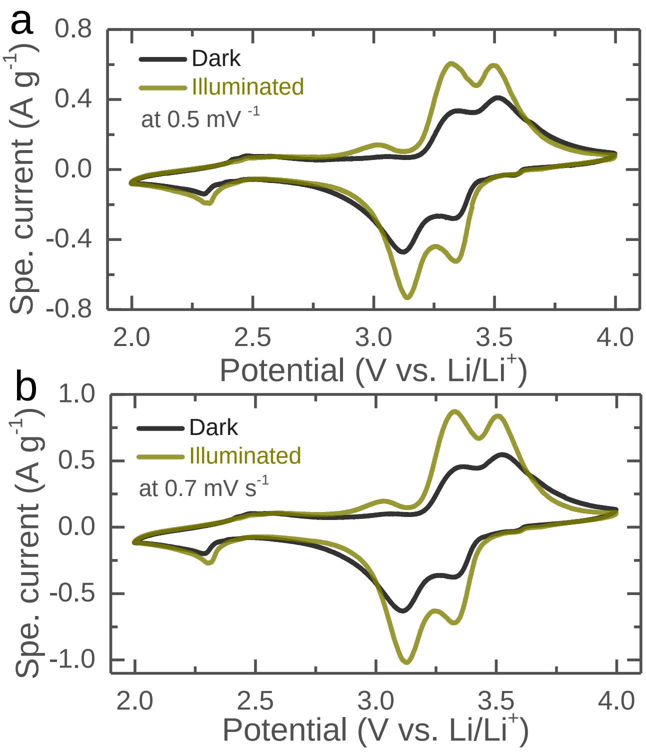

Figure S4. *(a,b) Comparative CVs at scans of 0.5 mV s^{-1} and 0.7 mV s^{-1} in dark and illuminated recorded in 1M LiTFSI.*

The comparative CV curves at scan rates of 0.5 mV s^{-1} and 0.7 mV s^{-1} in dark and illuminated (λ ~ 455 nm, intensity ~ 12 mW cm^{-2}) conditions tested in 1M LiTFSI also support capacity enhancements under illumination as compared to that of dark conditions because of the photosensitive characteristics of the photocathodes.

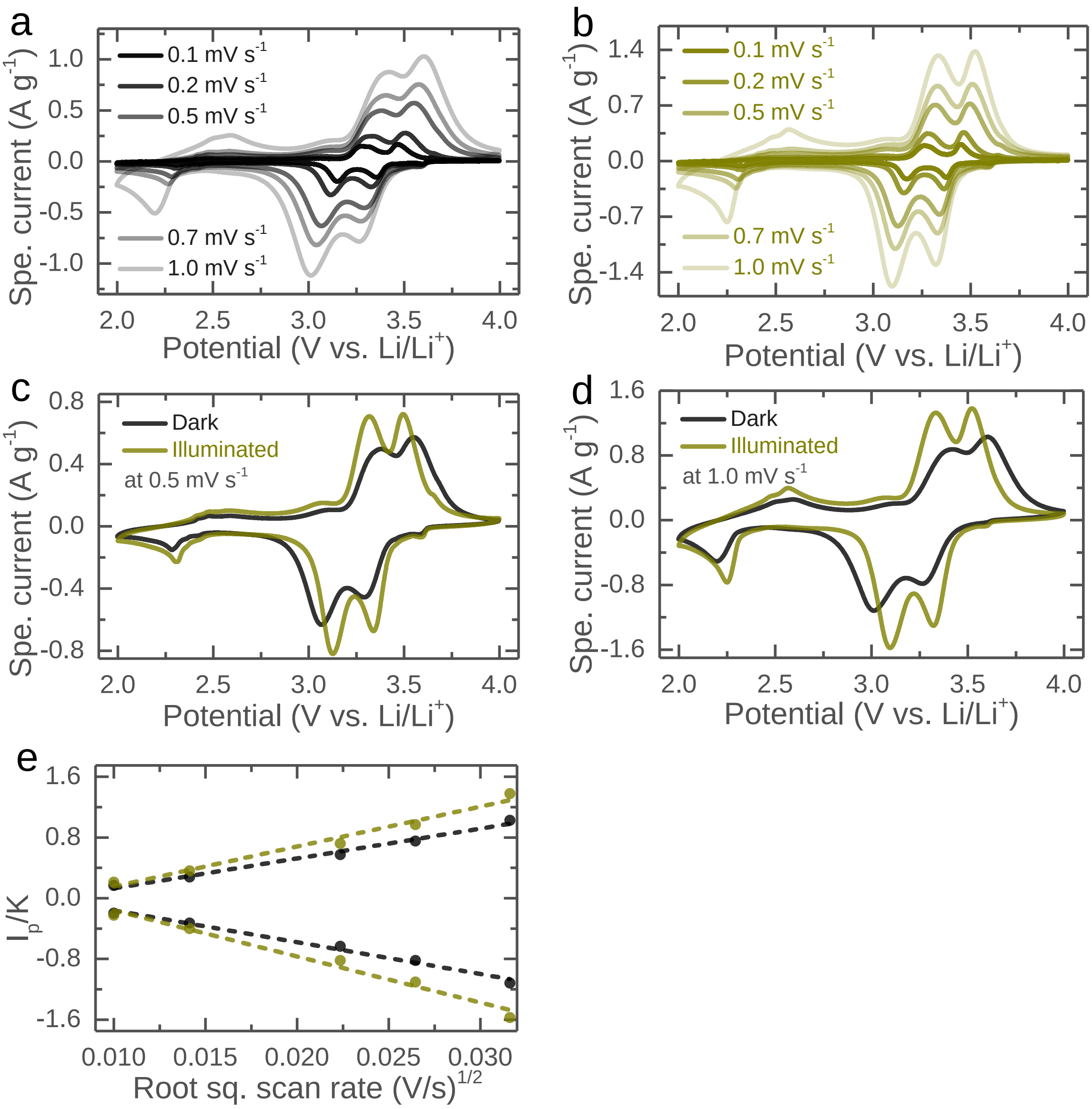

Figure S5. *(a,b) CVs of Photo-LIBs at different scans in dark and illuminated (λ ~ 455 nm, intensity ~ 12 mW cm*$^{-2}$*) conditions. (c,d) Comparative CVs at scans of 0.5 mV s*$^{-1}$ *and 1.0 mV s*$^{-1}$ *in dark and illuminated (λ ~ 455 nm, intensity ~ 12 mW cm*$^{-2}$*) conditions. (e) Comparative studies of diffusion constants in dark and illuminated conditions. These measurements tested in 5M LiTFSI.*

The dark and light illuminated CVs at scans of 0.1 to 1.0 mV s^{-1} measured in 5M LiTFSI of the Photo-LIBs shown in **Figure S5a**,**b**, where the comparative CVs at 0.5 mV s^{-1} (**Figure S5c**) and 1.0 mV s^{-1} (**Figure S5d**) show capacity enhancements under illumination. Further, ~ 45%

and ~ 34% enhancements in Li^+ diffusion constants observed in cathodic (peak centered at ~ 3.11 V) and anodic (peak centered at ~ 3.53 V) reactions.

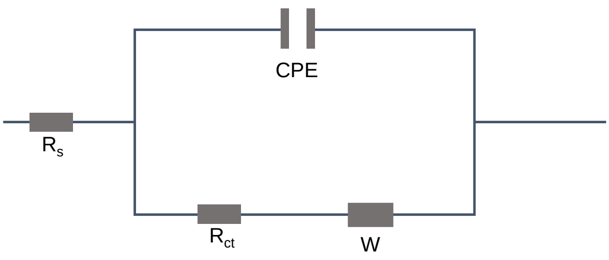

Figure S6. *Equivalent circuit for the Nyquist plots (Figure 3f) in dark and illuminated conditions, where, R_s represents the total resistance of the electrode, electrolyte, and separator, R_{ct} refers to the charge transfer resistance, CPE represents double layer capacitance corresponding to the semicircles, and W refers to the Warburg impedance.*

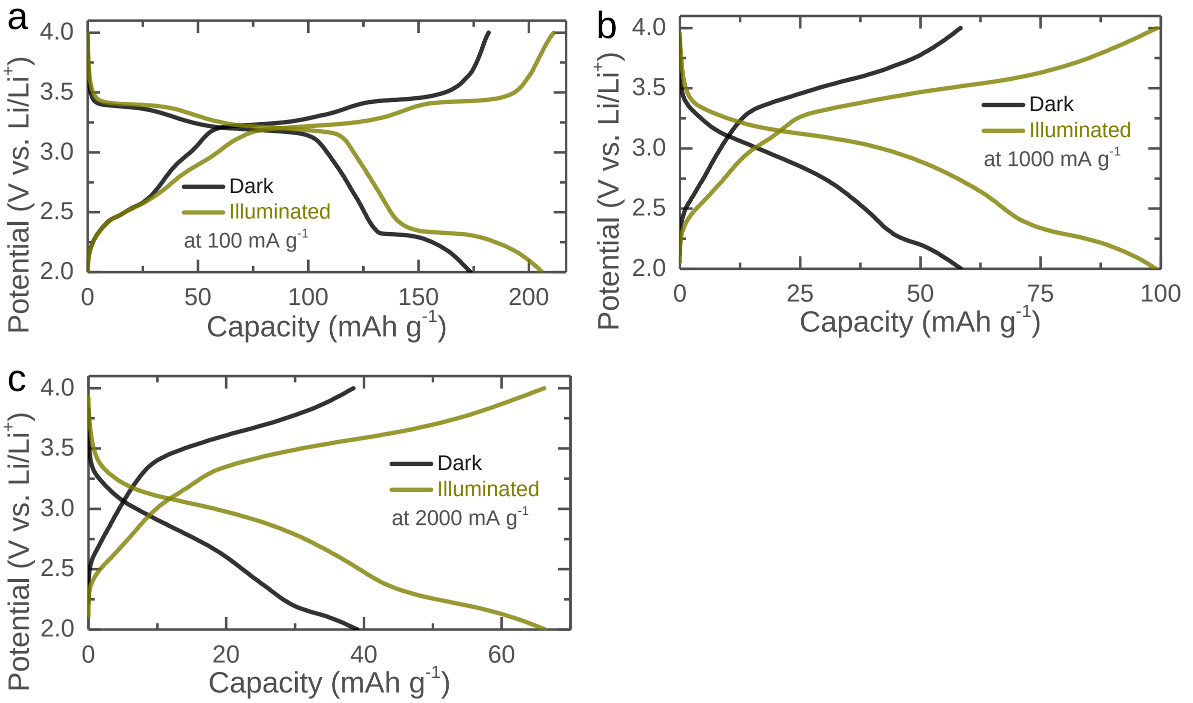

Figure S7. *(a-c) Comparative discharge-charge curves at specific currents of 100 mA g^{-1} (2nd cycle), 1000 mA g^{-1} (17th cycle) and 2000 mA g^{-1} (22nd cycle) in dark and illumination conditions tested in 1M LiTFSI electrolyte.*

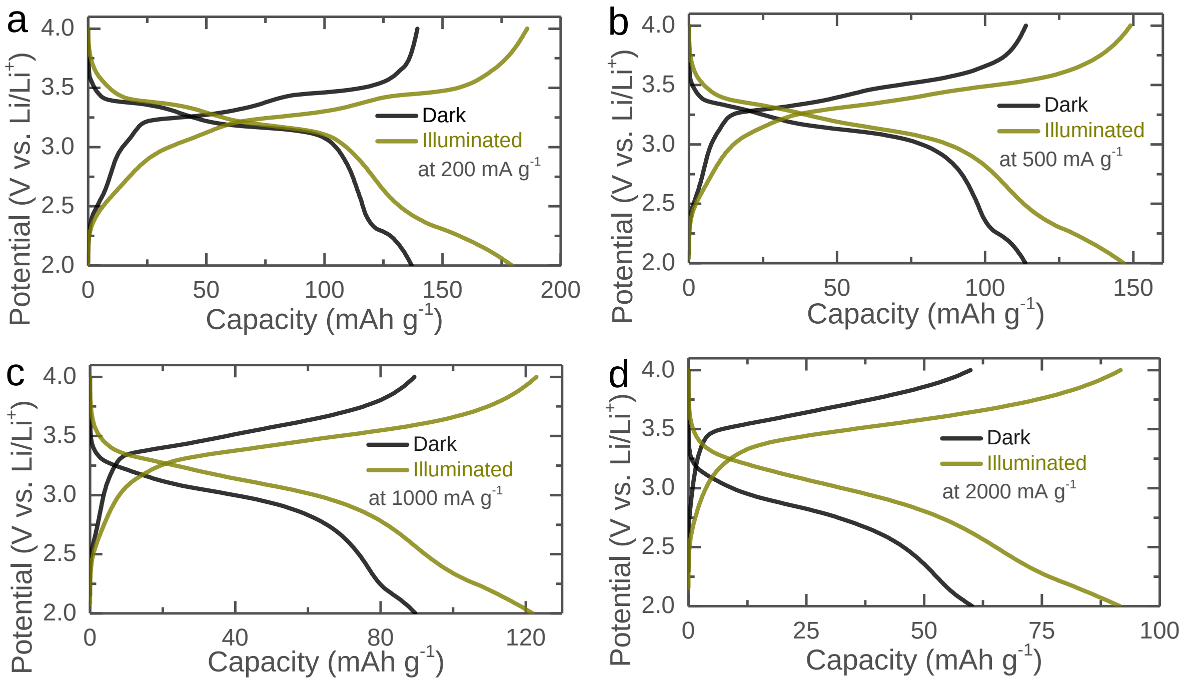

Figure S8. *Discharge-charge curves at specific currents of (a) 200 mA g^{-1} (7th cycle), (b) 500 mA g^{-1} (12th cycle), (c) 1000 mA g^{-1} (17th cycle) and (d) 2000 mA g^{-1} (22nd cycle) in dark and illumination conditions tested in 5M LiTFSI.*

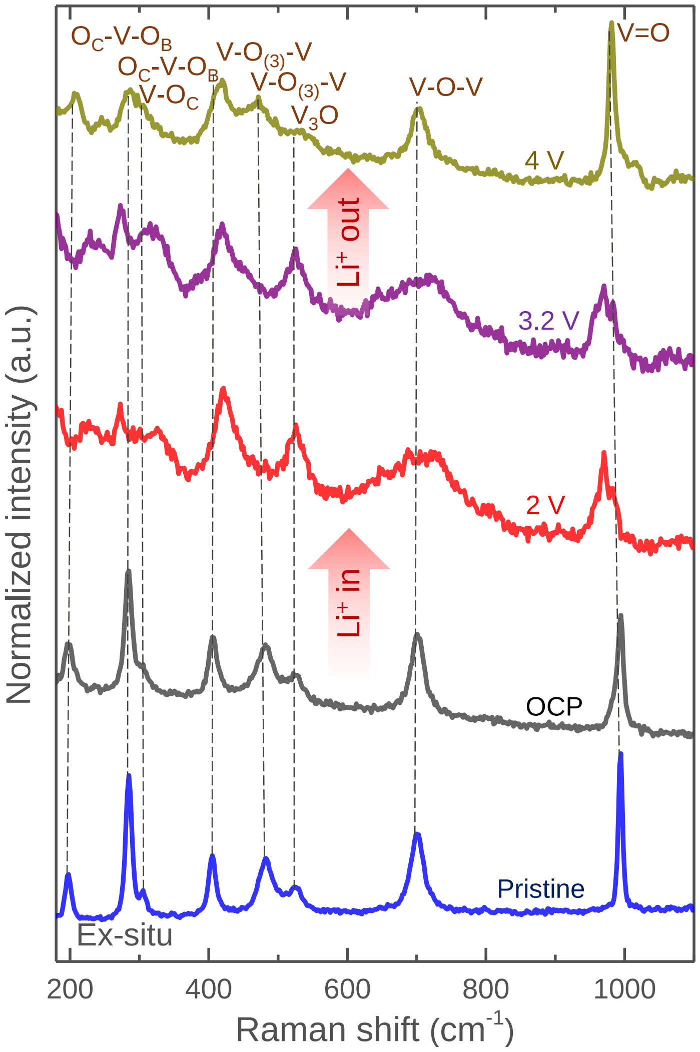

***Figure S9** Ex-situ Raman spectra of the photocathodes at different stages of discharge-charge (the right graph of **Figure 5a**).*

Figure S9 shows the *ex-situ* Raman spectra of the electrodes at different states of discharge and charge shown in **Figure 5a**. Under discharged to 2 V, the characteristic Raman shifts associated with the stretching of V – O – V bonds (~ 700 cm^{-1}), angle-bending of V – $O_{(3)}$ – V (~ 404 cm^{-1}) and bending vibration of V – O_C (~ 304 cm^{-1}) are shifted towards the higher Raman shift side, whereas the characteristic peaks of stretching vibration of vanadyl bond (V = O) bond (~ 994 cm^{-1}) and bond bending vibration of O_C – V – O_B (~ 282 cm^{-1}) are shifted towards lower Raman shift side. Moreover, V – $O_{(3)}$ – V symmetric stretching (~ 482 cm^{-1}) and bending vibration of O_C – V – O_B (~ 197 cm^{-1}) disappear under Li^+ insertion into

photocathodes. However, these characteristic Raman shifts are reversible when charged to the 4 V (Li^+ extraction).

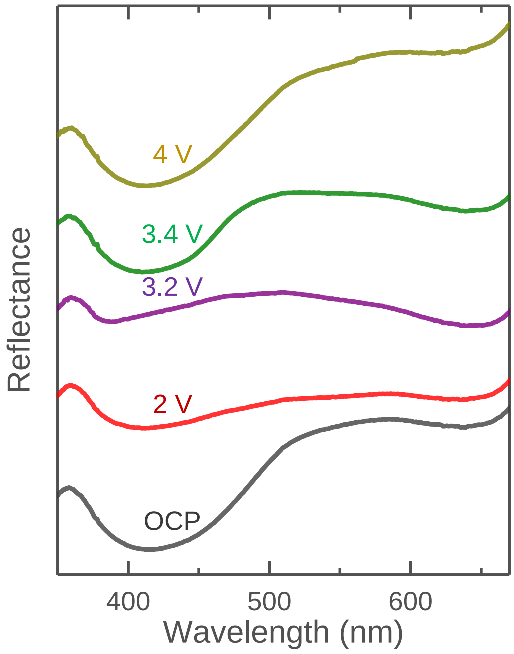

Figure S10. *The in-situ optical reflectance spectra of the photocathode at the respective states of discharge and charge marked in the discharge-charge curve of* ***Figure 5a****.*

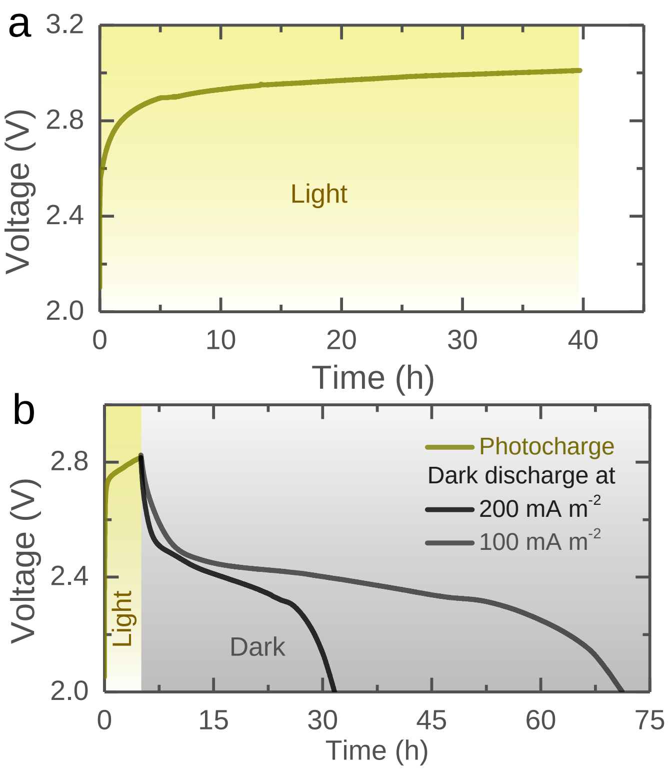

Figure S11. *(a) Long time photocharging response of the Photo-LIB under illumination in 1M LiTFSI. (b) Photocharge and discharges at different specific currents of 100 mA m*$^{-2}$ *and 200 mA m*$^{-2}$ *tested in 1M LiTFSI.*

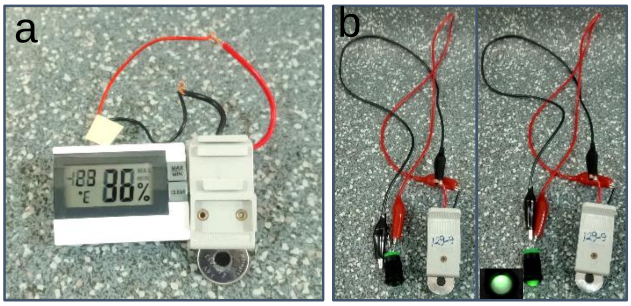

Figure S12. *(a,b) Digital photographs showing a 1.5 V Thermo-hygrometer and a green LED powered by a single Photo-LIB charged by light (λ ~ 455 nm, intensity ~ 12 mW cm*$^{-2}$*).*

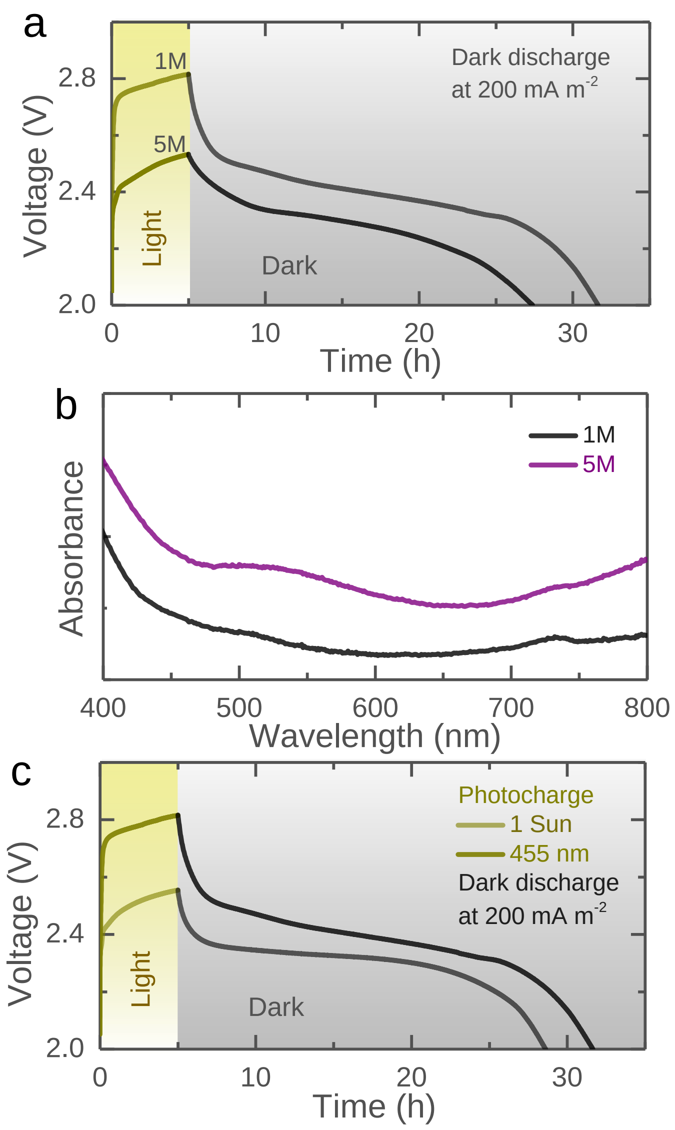

Figure S13. *(a) Photocharges and discharges (at 200 mA m^{-2}) of the Photo-LIBs with different LiTFSI electrolyte concentrations of 1M and 5M, respectively. (b) Absorption spectra of the 1M and 5M LiTFSI electrolytes. (c) Photocharge under 1 Sun and 455 nm illuminations and discharges at specific current of 200 mA m^{-2} tested in 1M LiTFSI.*

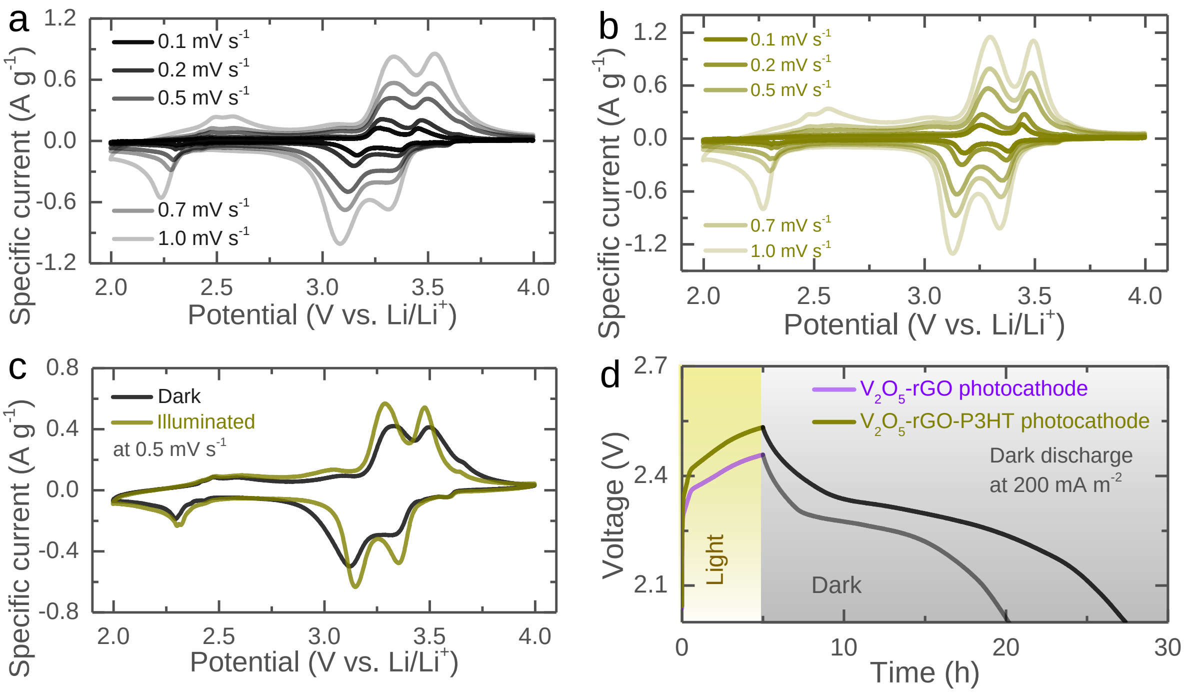

Figure S14. *(a,b) CVs at different scans (0.1 – 1.0 mV s^{-1}) in dark and illuminated of the P3HT free V_2O_5-rGO photocathodes based Photo-LIBs recorded in 5M LiTFSI electrolyte. (c) Comparative CVs at 0.5 mV s^{-1} in dark and illuminated of the V_2O_5-rGO photocathodes based Photo-LIBs. (d) Photocharges of the Photo-LIBs based on P3HT free V_2O_5-rGO and V_2O_5-P3HT-rGO photocathodes, which are tested in 5M LiTFSI electrolyte.*

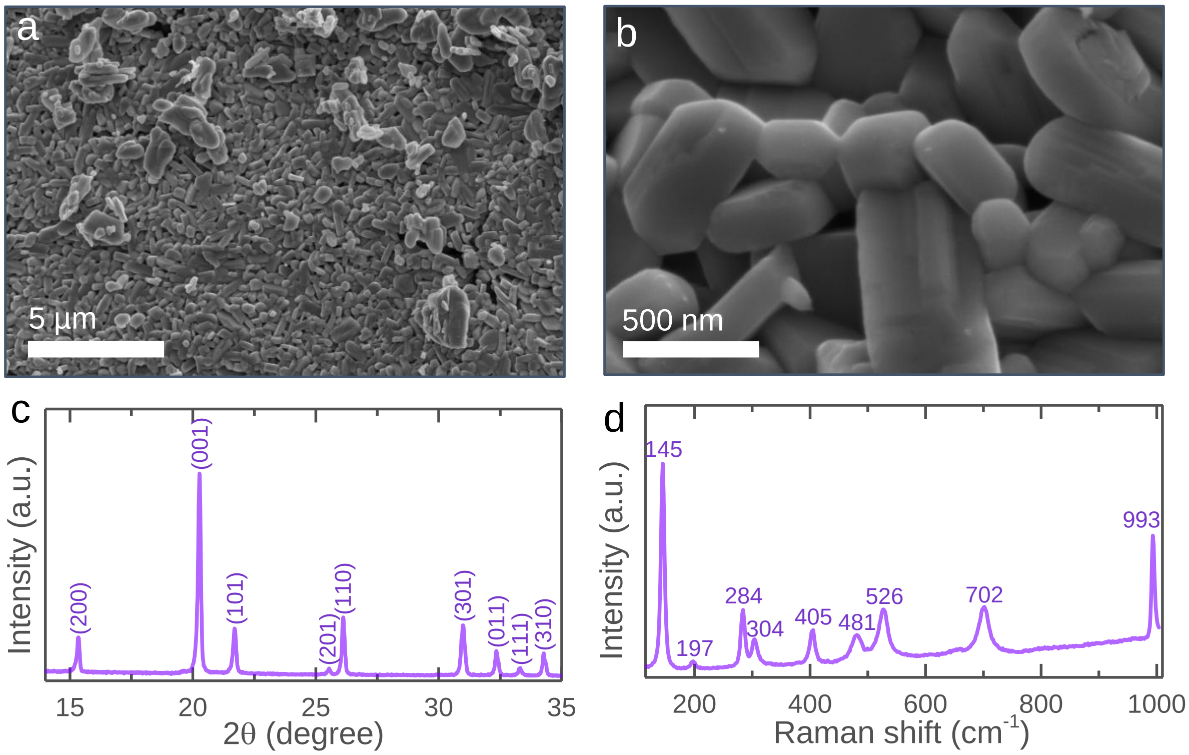

Figure S15. *(a,b) SEM images at low and high magnifications of* V_2O_5 *powder (primary particles) used for the synthesis of nanofibers. (c) XRD pattern of* V_2O_5 *powder belongs to orthorhombic* V_2O_5 *crystals structure with space group of Pmmn (59) (JCPDS card no: 03-065-0131). (d) Raman of the* V_2O_5 *powder.*

Efficiency calculation:

The photo-conversion efficiencies (η) of the Photo-LIB can be expressed as,

$$\eta = \frac{E_{out}}{E_{in}} \times 100\% = \frac{\frac{E}{A_1} \times A_1}{P_{in} \times t_{ph} \times A_{ph}} \times 100\%$$

Where, E (= 0.588 mWh for 455 nm illumination and 0.416 mWh for 1 Sun illumination) is the discharge energy at discharge specific current of 100 mA m^{-2}, A_1 (= 0.38 cm^2) is the active photocathode area, P_{in} (= 12 mW cm^{-2} for 455 nm illumination and 100 mW cm^{-2} for 1 sun illumination) is the illuminated light intensity, t_{ph}(= 5 h) represents photocharging time and

$A_{ph}(= 0.38\ cm^2)$ is the illuminated surface area. Based on this relation, the calculated photo-conversion efficiencies are ~ 2.6% for 455 nm illumination and ~ 0.22% for 1 sun illumination.

References

1. Ahmad, S.; George, C.; Beesley, D. J.; Baumberg, J. J.; De Volder, M. Photo-Rechargeable Organo-Halide Perovskite Batteries. *Nano Lett.* **2018**, *18,* 1856.

2. Chou, J. Y.; Lensch-Falk, J. L.; Hemesath, E. R.; Lauhon, L. J. Vanadium oxide nanowire phase and orientation analyzed by Raman spectroscopy. *J. Appl. Phys.* **2009**, *105*, 034310.

3. Zhou, B.; He, D. Y. Raman spectrum of vanadium pentoxide from density-functional perturbation theory. *J. Raman Spectrosc.* **2008**, *39*, 1475.

4. Zhai, T.; Liu, H.; Li, H.; Fang, X.; Liao, M.; Li, L.; Zhou, H.; Koide, Y.; Bando, Y.; Golberg, D. Centimeter-long V2O5 nanowires: from synthesis to field-emission, electrochemical, electrical transport, and photoconductive properties. *Adv. Mater.* **2010**, *22*, 2547.
